# Household HIV Testing Uptake among Contacts of TB Patients in South Africa

**DOI:** 10.1371/journal.pone.0155688

**Published:** 2016-05-19

**Authors:** Kavindhran Velen, James J. Lewis, Salome Charalambous, Liesl Page-Shipp, Flora Popane, Gavin J. Churchyard, Christopher J. Hoffmann

**Affiliations:** 1 The Aurum Institute, Johannesburg, South Africa; 2 The School of Public Health, University of Witwatersrand, Johannesburg, South Africa; 3 London School of Hygiene and Tropical Medicine, London, United Kingdom; 4 Advancing Care and Treatment for TB and HIV, MRC Collaborating Centre of Excellence, Johannesburg, South Africa; 5 Johns Hopkins University School of Medicine, Baltimore, United States of America; University of Malaya, MALAYSIA

## Abstract

**Background:**

In high HIV prevalence settings, offering HIV testing may be a reasonable part of contact tracing of index tuberculosis (TB) patients. We evaluated the uptake of HIV counselling and testing (HCT) among household contacts of index TB patients and the proportion of newly diagnosed HIV-infected persons linked into care as part of a household TB contact tracing study.

**Methods:**

We recruited index TB patients at public health clinics in two South African provinces to obtain consent for household contact tracing. During scheduled household visits we offered TB symptom screening to all household members and HCT to individuals ≥14years of age. Factors associated with HCT uptake were investigated using a random effects logistic regression model.

**Results & Discussion:**

Out of 1,887 listed household members ≥14 years old, 984 (52%) were available during a household visit and offered HCT of which 108 (11%) self-reported being HIV infected and did not undergo HCT. Of the remaining 876, a total of 304 agreed to HCT (35%); 26 (8.6%) were newly diagnosed as HIV positive. In multivariable analysis, factors associated with uptake of HCT were prior testing (odds ratio 1.6; 95% confidence interval [CI]: 1.1–2.3) and another member in the household testing (odds ratio 2.4; 95% CI: 1.7–3.4). Within 3 months of testing HIV-positive, 35% reported initiating HIV care.

**Conclusion:**

HCT as a component of household TB contact tracing reached individuals without prior HIV testing, however uptake of HIV testing was poor. Strategies to improve HIV testing in household contacts should be evaluated.

## Introduction

Achieving maximum health and prevention benefits from antiretroviral therapy (ART) requires crossing a first threshold: diagnosis of HIV-infection. In 2014 UNAIDS proposed the 90-90-90 target [1] proposing that by 2020 (1) 90% of all people living with HIV will know their status; (2) 90% of all people diagnosed HIV infection will receive sustained antiretroviral therapy (ART); and (3) 90% of all people receiving ART will have viral suppression. National and international funding has made HIV testing widely available in many of the parts of Africa most affected by HIV, including South Africa. In 2010 South Africa launched an ambitious campaign to perform 15 million HIV tests [2, 3]. Despite impressive HIV counselling and testing (HCT) delivery in South Africa, 62% of HIV-infected men and 45% of HIV-infected women are unaware of their HIV status [3]. Efforts to achieve test and treat goals, reduce late presentation and associated morbidity, and achieve the UNAIDS 90-90-90 targets will require reaching more individuals earlier in the course of HIV infection.

Several models of HCT have been used, including facility-based, stand-alone, mobile, and household [4, 5]. The World Health Organization HCT guidelines currently recommend a mixed approach, deploying multiple strategies to reach individuals, especially those at highest risk for HIV [6]. Evidence from household testing has reported a high prevalence of undiagnosed HIV [7] and may provide a means to overcome two barriers: access to testing sites and perception of a low risk for HIV and low value in testing [8–10].

Household contact tracing of known index TB patients is an important TB prevention strategy in high burden settings [11] and provides an ideal opportunity to offer HCT during household TB contact screening. Identifying household contacts with HIV may enable more people to link into care for ART. We evaluated HCT uptake during household contact tracing among contacts of index TB patients, assessed for factors associated with the uptake of testing, and determined entry-into-care for HIV within three months of testing.

## Methods

### Ethics Statement

All aspects of this study were conducted according to the principles expressed in the Declaration of Helsinki. Human subjects research approval, including approval of the consent process and consenting documents, was granted by the University of the Witwatersrand Medical Human Subjects Research Ethics committee and the London School of Hygiene and Tropical Medicine Research Ethics Committee. Written informed consent was obtained from all participants aged 18 or older. Written (signed) assent for research participation was obtained from all participants aged 14–17 years old; following obtaining assent, written (signed) informed consent was obtained from a guardian for those participants.

### Setting

*The Inhibit-TB study* was a cluster randomized trial designed to determine the effectiveness of adding isoniazid preventative therapy and point-of-care CD4 count testing in a contact tracing program based in rural and urban regions of South Africa. Index TB patients were recruited at public health clinics following their diagnosis with pulmonary TB. The households of consenting index TB patients were randomized to receive household contact tracing for TB and HCT plus one of three types of additional subsequent HIV or TB prevention related care packages. In addition to providing a listing of household members residing with them, the index TB patients also provided the most suitable times for which the household contacts would be available for visitation. Each household was then visited up to 3 times to accommodate the most suitable times of household contacts. We offered HCT to household contacts that were ≥14 years old because they were able to consent to HIV testing without parental consent. In addition, the prevalence of undiagnosed HIV is very low among children <14 years old in South Africa thus we did not offer testing to that age group [3]. We limited this secondary analysis to the standard of care arm to eliminate concerns that additional services may have influenced HCT uptake.

### HIV counselling and testing

HCT was conducted according to the National HIV Counselling and Testing Policy Guidelines (2010) [12] with the provision of pre- and post-test counselling and written consent for HIV-testing. HCT was performed with particular consideration on ensuring confidentiality and privacy during and after testing. A room inside the household or an area outside the dwelling was used to allow for a private counselling sessions. Point-of-care testing was done using the Alere Determine^™^ HIV-1/2 test. If the initial test was positive, confirmation was done using the point-of-care Trinity Biotech Uni-Gold^™^ Recombigen^®^ HIV-1/2 confirmatory test. In instances where a participant was HIV negative on the confirmatory test after testing HIV-positive on the screening test, a venous blood sample was taken for laboratory ELISA analysis.

Participants who tested HIV-positive were provided referral letters to attend the health facility of their choice for HIV care. Contacts 14–17 years old who tested HIV-positive were informed of their status in the presence of the parent/guardian issuing the initial consent for study participation; this was followed by post-test counselling and a visit from a social worker. A structured questionnaire was administered to all contacts offered HCT regardless of whether they eventually tested or not. Follow-up contact was attempted for all participants who tested HIV positive to verify clinic attendance.

### Data analysis

We determined the proportion of the total household (≥14 years old) present at the time of the household visit, the proportion present not having tested previously or with prior HIV-negative results, and the proportion reporting HIV-positive status (repeat HCT unnecessary). The index TB household member was not included in the household testing because he or she received clinic-based provider initiated testing and counselling as part of TB care. Univariable and multivariable analysis was done using a random effects logistic regression model for HCT uptake to determine significant factors associated with uptake. Factors with a p<0.2 were included in multivariable model building. We took into account the effect of household and district as random effects in the model. Factors analysed in the model included age, gender, education, income, mobile phone ownership, number of contacts living in the household, history of ever testing for HIV, index TB patient HIV status, and index TB patient on ART or cotrimoxazole. Entry into HIV care was defined as any self-reported evidence of visiting a health facility subsequent to HIV diagnosis and 1) registered and give a patient number/card; or 2) blood drawn for CD4 testing; or 3) started on ART.

## Results

### Baseline characteristics

Between January 1, 2013 and October 29, 2014, in the standard of care arm, a convenience sample of 754 index TB patients agreed to participate representing 754 households. There were a total of 2,986 individuals listed as household members by the index TB patient, in addition to him or her, on enrolment. Of these, 1,887 were ≥14 years old, 909 were <14 years old, and 190 had an unrecorded age. Of the 1,887 ≥14 years old, 775 were male (41.1%) and 1,112 were female (58.9%). During household visits, 984 (52%) household members ≥14 years old were present and enrolled in the study. The median age of these participants was 30 years (inter-quartile range [IQR]: 21, 51); 351 (35.7%) were men and 631 (64.3%) were women (two had an unrecorded gender).

**Fig 1:**
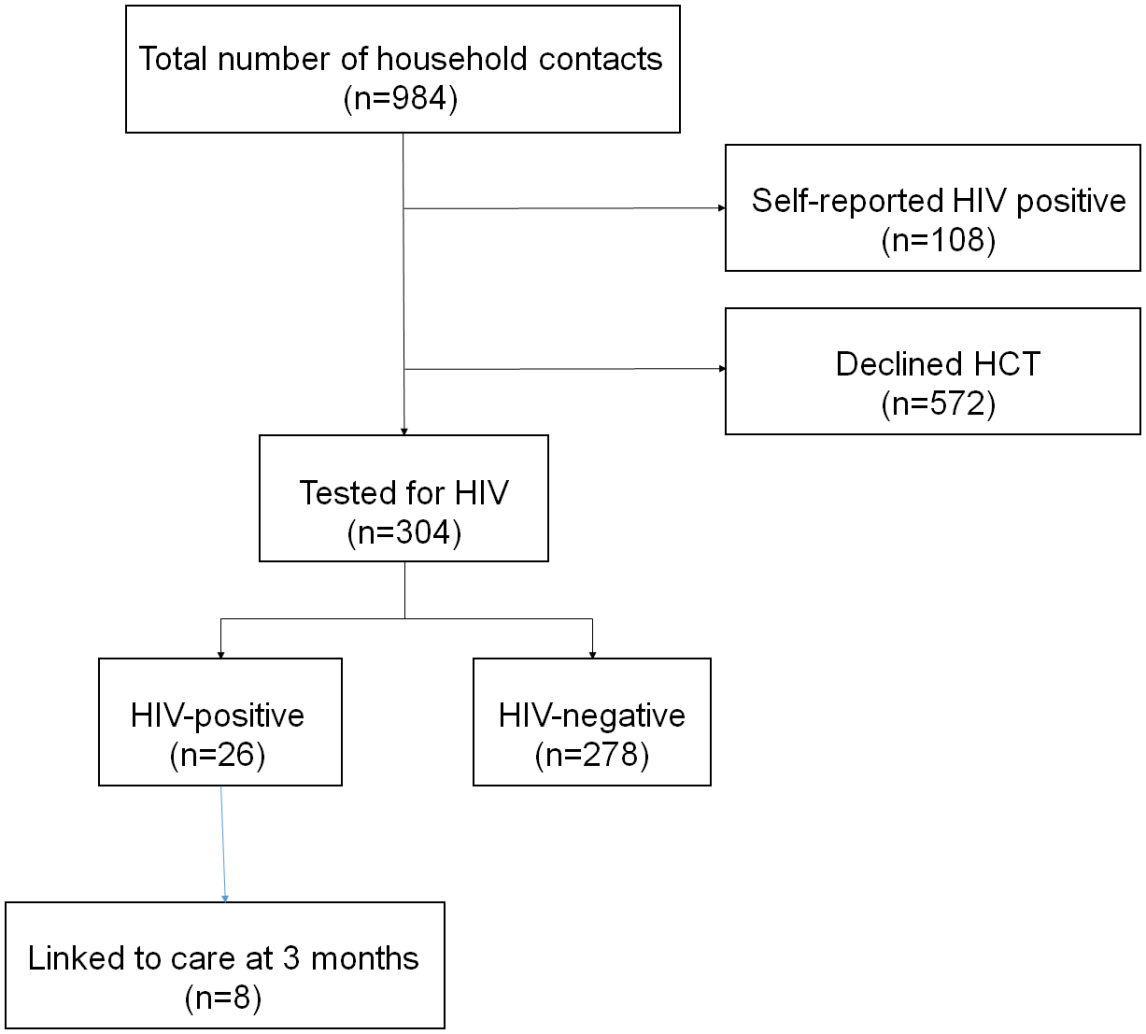
Flow diagram of household contacts present at home and screened for HIV.

Of the 984 household members aged 14 years or older, 108 (11%) reported being HIV-infected (Fig 1). Of those not reporting being HIV-infected (n = 876), 304 (35%) agreed to HCT (Fig 1). Women were similarly distributed among those agreeing to and declining HCT (63% versus 64%; Table 1). The age distribution was similar between those undergoing and those declining HCT. Among those undergoing HCT, 64% reported a history of prior HCT compared to 49% among those who declined testing.

**Table 1:** Demographic characteristics of household contacts who were offered an HIV test (n = 876).

Characteristic	N (column %)	Tested for HIV	Unadjusted OR	Adjusted OR
		n (row %)	(95% CI) *	(95%CI) *
Gender			p = 0.504	p = 0.229
Men	328 (37.4%)	109 (33.3%)	1	1
Women	548 (62.6%)	195 (35.6%)	1.13 (0.79, 1.62)	1.25 (0.87, 1.79)
Age (years)			p = 0.9	p = 0.2
14–24	335 (38.2%)	112 (33.4%)	1	1
25–39	201 (22.9%)	68 (33.8%)	1.05 (0.66, 1.68)	0.73 (0.44, 1.21)
≥40	340 (38.8%)	124 (36.5%)	1.06 (0.72, 1.58)	1.15 (0.70, 1.88)
District			p<0.001	p = 0.009
Urban/Semi-urban	655 (74.8%)	263 (40.2%)	1	1
Rural	221 (25.2%)	41 (18.6%)	0.23 (0.13, 0.41)	0.52 (0.31, 0.86)
Level of education			p = 0.02	p = 0.03
≤ Grade 7	224 (25.6%)	63 (28.1%)	1	1
Grade 8–11	413 (47.1%)	153 (37.0%)	1.65 (1.04, 2.62)	1.65 (1.04, 2.61)
Grade 12	174 (19.9%)	72 (41.4%)	1.97 (1.13, 3.44)	1.77 (1.00, 3.13)
Tertiary	50 (5.7%)	13 (26.0%)	0.82 (0.34, 1.96)	0.78 (0.33, 1.84)
Missing	15 (1.7%)	3 (20.0%)		
Employment status			p = 0.008	p = 0.6
Formal employment	100 (11.4%)	49 (49.0%)	1	1
Self-employment	21 (2.4%)	10 (47.6%)	1.06 (0.31, 3.61)	1.19 (0.43, 3.34)
Government grant	197 (22.5%)	63 (32.0%)	0.49 (0.26, 0.93)	0.75 (0.41, 1.37)
Other income	64 (7.3%)	24 (37.5%)	0.64 (0.28, 1.47)	0.90 (0.43, 1.88)
Student	170 (19.4%)	38 (22.4%)	0.29 (0.15, 0.57)	0.51 (0.23, 1.16)
No income	299 (34.1%)	112 (37.5%)	0.56 (0.31, 1.01)	0.83 (0.43, 1.61)
Unknown	25 (2.9%)	8 (32.0%)		
Level of monthly income			p = 0.05	p = 0.2
<$100	457 (52.2%)	164 (35.9%)	1	1
≥$100	227 (25.9%)	97 (42.7%)	1.50 (1.00, 2.23)	1.42 (0.87, 2.32)
Unknown	192 (21.9%)	43 (22.4%)		
Ownership of a mobile phone			p = 0.5	
No	39 (4.5%)	10 3.3%)	1	
Yes	835 (95.5%)	294 (96.7%)	1.40 (0.51, 3.61)	
History of previous HIV test			p<0.001	p = 0.01
No	400 (45.7%)	109 (27.3%)	1	1
Yes	476 (54.3%)	195 (41.0%)	2.10 (1.43, 3.08)	1.59 (1.10, 2.32)
Number of contacts in household			p = 0.5	
1–4	693 (79.1%)	242 (79.6%)	1	
5–8	159 (18.2%)	49 (16.1%)	0.75 (0.41, 1.40	
>8	24 (2.7%)	13 (4.3%)	1.95 (0.37, 10.19)	
Another household member tested today			p<0.001	p<0.001
No	506 (57.8%)	123 (24.3%)	1	1
Yes	370 (42.2%)	181 (48.9%)	2.71 (2.02, 3.63)	2.40 (1.71, 3.37)
HIV status of index TB patient			p = 0.4	
Positive	246 (28.1%)	76 (30.9%)	1	
Negative	539 (61.5%)	189 (35.1%)	1.02 (0.62, 1.69)	
Unknown	91 (10.4%)	39 (42.9%)	1.58 (0.74, 3.34)	
Index TB patient on ART or Cotrimoxazole			p = 0.7	
No	574 (65.5%)	203 (66.8%)	1	
Yes	302 (34.5%)	101 (33.2%)	0.92 (0.59, 1.43)	

* adjusted for district and with a random effect for the household

In a multivariable model, factors that were associated with the uptake of HCT were a history of previous HIV testing (odds ratio was 1.6 (95% CI: 1.1–2.3), another member in the household testing (odds ratio 2.4 (95% CI: 1.7–3.4), increased level of education, and older age (Table 1).

Of the 304 household members who underwent HCT, 26 (8.6%) were newly diagnosed as HIV positive and 278 were HIV negative (Fig 1). A 3-month follow-up was successfully completed for 23/26 (88%) of the newly diagnosed HIV-infected household members; 8 (35%) reported entry-into-care.

## Discussion

Among all household members ≥14 years of age present during household visits and without known HIV infection uptake of HCT was 35%. Another study from South Africa reported that 55% of index TB patient contacts tested for HCT when offered [7]. In contrast, other household-based HCT studies, not provided as part of TB contact tracing, have achieved higher uptake [9, 13, 14]; ranging from 69% to 98% [15]. The cause of the difference is unclear. It is plausible that community outreach and engagement activities that were part of the dedicated household HCT activities and are not feasible in TB contact tracing were a vital part of achieving a high uptake. The uptake of HCT by only 35% of household contacts highlights the challenge of integrated or “bundled” service delivery and a trade-off of lower uptake when delivering HCT as an add-on to other services compared to dedicated HCT delivery. Further HIV testing directed community or household engagement could be explored to increase HCT uptake.

We found specific characteristics associated with higher HCT uptake. There was an association within a household regarding HCT uptake suggesting that interpersonal relationships and household perceptions around HIV may have affected HCT uptake. One explanation for our finding may be that when HCT occurs in the household it is a household-level decision as well as an individual-level decision [5, 16, 17].

We diagnosed HIV among 26 individuals previously unaware of their status. Of these, 35% reported initiating HIV care. This proportion is consistent with some prior reports from mobile and household HCT in which 10 to 35% entered care within 3 months [18, 19]. It is less than reported from a clinic-based HCT study in which 72% were linked to care and much less than the 97% visiting an HIV clinic within 12 months of HCT in a household HCT study with a comprehensive community-based component [20, 21]. Our findings suggest that HIV diagnosis during household screening leads some, but not all, individuals to access care. We assume that multiple individual and clinic barriers contributed to low care engagement [22]. Additional interventions such as point-of-care CD4 testing (POC CD4), home-based ART initiation, and streamed-lined HIV care service delivery may improve timely entry-into-care [23].

This study has the important strength that it is a real-world examination of the success of bundling HCT with household TB contract tracing. In addition, it was conducted in rural and urban areas in two distinct provinces in South Africa. There are also several limitations to consider. First, we tested less than half of participants with unknown HIV status thus we are not able to estimate the overall HIV prevalence. Second, we used self-report regarding HIV care entry and were unable to contact all participants who had tested HIV positive. It is possible that participants over-reported entry-into-care for HIV. Lastly, we did not offer testing to individuals who were <14 years old. It is possible that we missed undiagnosed HIV in this age group. However, HIV positive rates in this population group are currently very low in South Africa [3].

## Conclusions

Adding HIV testing to household TB contact screening may be a way to increase targeted HIV testing through bundling with already ongoing activities. However further operational research is needed to identify ways to consistently achieve high uptake. Provisions to avoid disclosure (including possibly communicating results at a later time), generating demand (through incentives or health education), or to normalize household HIV testing (through community-level activities) may help to achieve a higher uptake of HCT. Increasing timely entry into care after testing HIV-positive may require other strategies such as offering home-based ART initiation [24] or other approaches to community-based delivery of care. If we hope to reach 90-90-90 targets, innovation is needed for both increased targeted HIV testing and improving entry and retention in care.

## Supporting Information

S1 File Household HCT Data. (CSV)
